# Splenic Artery Aneurysm Presenting as Extrahepatic Portal Vein Obstruction: A Case Report

**DOI:** 10.1155/2011/908529

**Published:** 2011-08-01

**Authors:** T. P. Elamurugan, S. Suresh Kumar, R. Muthukumarassamy, Vikram Kate

**Affiliations:** Department of Surgery, Jawaharlal Institute of Postgraduate Medical Education and Research (JIPMER), Puducherry 605006, India

## Abstract

Splenic artery aneurysms are the most common visceral aneurysm occuring predominantly in females. They are usually asymptomatic, and the symptomatic presentation includes chronic abdominal pain of varied severity or an acute rupture with hypotension. Splenic artery aneurysm causing extrahepatic portal hypertension is very rare and is due to splenic vein thrombosis that develops secondary to compression by the aneurysm. We report one such rare presentation of splenic artery aneurysms in a pregnant female with the features of EHPVO (variceal bleed, hypersplenism) treated by splenectomy along with excision of the aneurysm.

## 1. Introduction

Splenic artery aneurysms (SAAs) are the most common visceral aneurysm accounting for up to 60% in the reported series [1]. The incidence of splenic artery aneurysm is 0.01–0.2%, occuring more commonly in females especially during pregnancy with a female to male ratio of 4 : 1. Up to 80% of the SAA are detected in asymptomatic patients on routine evaluation for other problems [1]. Symptomatic presentation includes chronic abdominal pain of varied severity or an acute rupture in the peritoneal cavity causing hemorrhagic shock and presenting with hypotension. Atypical presentation of the SAA reported in literature includes acute massive upper gastrointestinal bleed due to rupture of aneurysm in to the stomach or pancreas and lower gastrointestinal bleeding due to rupture in to the colon [2–4]. SAA causing EHPVO and presenting with features of portal hypertension is extremely rare, Only few cases have been reported in the literature [5–7]. Here we report an unusual presentation of splenic artery aneurysm causing extrahepatic portal venous obstruction (EHPVO) and presenting with upper gastrointestinal bleed and hypersplenism. 

## 2. Case Report

A 22-year-old lady presenting to the antenatal clinic at 12 weeks of gestation was found to have pallor and massive splenomegaly. Complete haemogram profile showed pancytopenia with haemoglobin 9.2 gm%, total count 1500/cm^3^, and platelet count 30,000/cm^3^. Liver function test was normal. Bone marrow biopsy revealed hypercellular marrow. Ultrasound abdomen showed enlarged spleen of size 18 cms with no focal lesions and a normal liver. Portal vein was dilated, and splenic vein was tortuous with thrombus in the distal part. Doppler study of portal system revealed dilated portal vein measuring 2 cm with eccentric thrombus and a decreased maximum flow velocity of around 15 cm/sec. Splenic vein was also dilated and tortuous with maximum diameter of 3 cm in the distal part with a concentric thrombus. Hepatic vein and intrahepatic veins were patent and showed normal flow pattern. Upper GI endoscopy showed three columns of grade II esophageal varices with moderate portal gastropathy. With these investigations the patient was diagnosed as a case of extrahepatic portal venous obstruction. Her pregnancy was continued under close monitoring, and she delivered at term by caesarian section which was done for foetal distress. Massively enlarged spleen was documented intraoperatively during caesarian section. Postpartum period was uneventful. 

Patient was lost to followup after delivery, and she presented 7 months postpartum to the emergency medical services with acute hematemesis. UGI endoscopy showed 3 columns of grade III esophageal varices with portal gastropathy. Endoscopic variceal ligation was done for esophageal varices. Investigations showed severe anemia with Hb of 5.4 gm%, total leukocyte count of 750/cm^3^, and platelet count of 11,000/cm^3^. Liver function tests were normal. Patient was planned for splenectomy to correct hypersplenism and devascularization procedure for portal hypertension.

**Figure 1:**
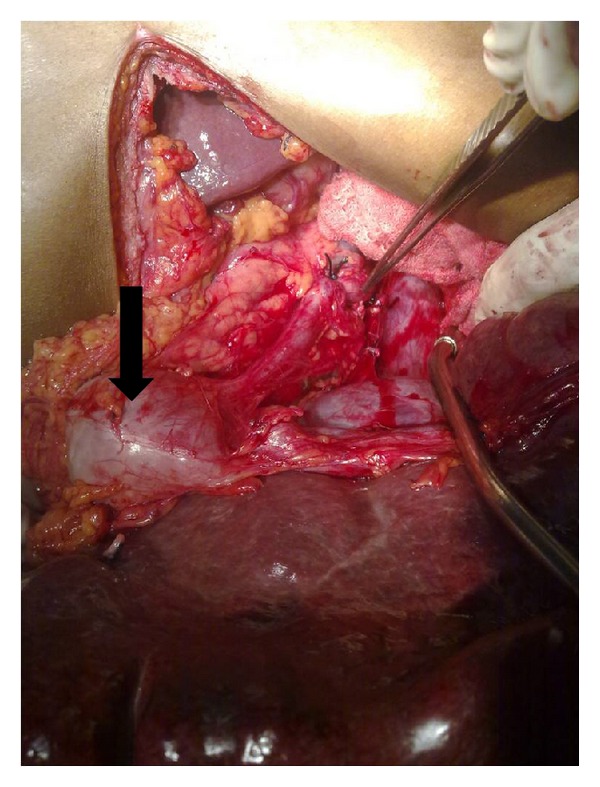
Intraop picture showing splenic artery aneurysm (arrow).

**Figure 2:**
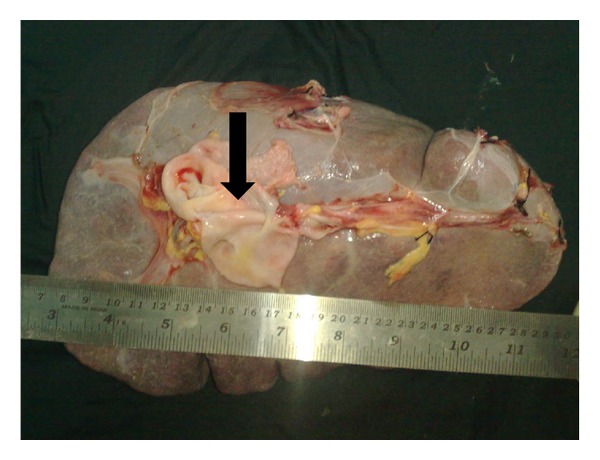
Specimen of spleen and aneurysm which was cut open (arrow).

At laparotomy, spleen was massively enlarged measuring roughly 25 × 15 cm, and an aneurysm of the splenic artery of size approximately 10 × 8 cm was present 2 cm proximal to the splenic hilum compressing the splenic vein (Figure 1). Splenic vein was found dilated and tortuous with multiple prominent collaterals in the lienorenal and splenocolic ligament. No collaterals were seen at liver hilum and portal triad. Liver and other organs were normal. Splenectomy along with resection of the aneurysm was carried out (Figure 2). Postoperatively patient made uneventful recovery. Counts normalized on day 3. On 3-month followup patient was pregnant again with normal blood counts, and varices have regressed to grade I with no portal gastropathy.

## 3. Discussion

Splenic artery aneurysms (SAAs) are the most common visceral aneurysm accounting for up to 60% [1] and have an incidence of 0.01–0.2%. Women are affected four times more commonly by SAA and often during pregnancy and childbearing years due to endocrine changes. Splenic artery aneurysm is usually single and ≤2 cm in size, whereas giant aneurysms (diameter > 2.5 cm) like in our patient are very rare [8]. The usual location of splenic artery aneurysm is at the mid or distal portion of the splenic artery, frequently found at the arterial bifurcation. In our patient aneurysm was located in the distal part of the splenic artery just proximal to the splenic hilum. Splenic artery aneurysms are asymptomatic in up to 80% of the patients, detected incidentally during other diagnostic imaging investigations [9]. In the present case investigations were suggestive of only an EHPVO when the patient was worked up for splenomegaly. SAA was detected only intraop when we opened the patient to do splenectomy (as she had hypersplenism). Symptomatic presentation ranges from chronic abdominal pain of varied severity to an acute rupture with hypotension, hemorrhagic shock, and a sudden collapse which occurs in 3–10% [1]. Pregnancy carries high risk of splenic artery rupture due to increased size because of hormonal influence. In the present case the patient was asymptomatic and had no clinical features suspicious of SAA except for splenomegaly. 

SAA causing EHPVO is extremely rare; only few cases have been reported in the literature [5–7]. The pathogenesis of extrahepatic portal venous obstruction is due to compression on the splenic vein leading to stasis and thereby predisposing to development of thrombosis. Isolated splenic vein thrombosis usually leads to sinistral portal hypertension. In our case thrombosis was found extending into portal vein leading to features of extrahepatic portal venous obstruction. The synchronous thrombus in the portal vein probably would have been an extension of the thrombus from the splenic vein. In such cases the presentation will be similar to that of the features of EHPVO which may include variceal bleeding, splenomegaly, and features of hypersplenism as in the present case. 

Treatment of SAA includes aneursyectomy with or without splenectomy and nonoperative endovascular techniques. Splenic artery aneurysm presenting with atypical symptoms of EHPVO as like our patient, splenectomy with excision of the aneurysm, excludes the risk of future aneurismal rupture and curative for the symptoms of EHPVO [5, 6].

## 4. Conclusion

Portal hypertension due to extrahepatic portal venous obstruction can occur in patients with splenic artery aneurysm secondary to compression on splenic vein causing thrombosis. Resection of the aneurysm along with splenectomy is curative in this condition.
